# Identification and Functional Characterization of Two Executioner Caspases in *Crassostrea gigas*

**DOI:** 10.1371/journal.pone.0089040

**Published:** 2014-02-13

**Authors:** Tao Qu, Baoyu Huang, Linlin Zhang, Li Li, Fei Xu, Wen Huang, Chunyan Li, Yishuai Du, Guofan Zhang

**Affiliations:** 1 Institute of Oceanology, Chinese Academy of Sciences, Qingdao, China; 2 University of Chinese Academy of Sciences, Beijing, China; McGill University, Canada

## Abstract

Caspase-3 and caspase-7 are two key effector caspases that play important roles in apoptotic pathways that maintain normal tissue and organ development and homeostasis. However, little is known about the sequence, structure, activity, and function of effector caspases upon apoptosis in mollusks, especially marine bivalves. In this study, we investigated the possible roles of two executioner caspases in the regulation of apoptosis in the Pacific oyster *Crassostrea gigas*. A full-length capase-3–like gene named *Cgcaspase-3* was cloned from *C.gigas* cDNA, encoding a predicted protein containing caspase family p20 and p10 domain profiles and a conserved caspase active site motif. Phylogenetic analysis demonstrated that both Cgcaspase-3 and Cgcaspase-1 may function as effector caspases clustered in the invertebrate branch. Although the sequence identities between the two caspases was low, both enzymes possessed executioner caspase activity and were capable of inducing cell death. These results suggested that Cgcaspase-3 and Cgcaspase-1 were two effector caspases in *C. gigas*. We also observed that nucleus-localized Cgcaspase-3, may function as a caspase-3–like protein and cytoplasm-localized Cgcaspase-1 may function as a caspase-7–like protein. Both *Cgcaspase-3* and *Cgcaspase-1* mRNA expression increased after larvae settled on the substratum, suggesting that both caspases acted in several tissues or organs that degenerated after oyster larvae settlement. The highest caspase expression levels were observed in the gills indicating that both effector caspases were likely involved in immune or metabolic processes in *C. gigas*.

## Introduction

Apoptosis, the process of programmed cell death that occurs in multicellular organisms, is characterized by a number of biochemical events leading to morphological changes – including chromosomal DNA fragmentation, chromatin condensation, cell shrinkage, and formation of apoptotic bodies [1], [2]. Although, apoptosis may adversely affect the function of multiple tissues and organs (and even the survival of animals under some pathophysiological conditions), it also plays important roles in many physiological processes, including normal tissue and organ development and homeostasis [3], [4].

The biochemical events of apoptosis are performed by a family of cysteine proteases known as caspases (cysteinyl aspartate-specific proteinase) [5]. In mammals, the caspase gene family consists of at least 13 members divided into two groups: (1) caspase-1, 4, 5, 11, 12 and 14 play a role in inflammatory responses; and (2) caspase-2, 3, 6, 7, 8, 9 and 10 participate in apoptosis. Apoptosis-participating caspases are further divided into initiator caspases (caspase-8, 9, 10) and effector caspases (caspase-3, 6, 7). Effector caspases usually consist of only one small prodomain and function downstream of initiator caspases. Once activated by initiator caspases, effector caspases cleave diverse intracellular substrates, ultimately resulting in apoptosis [6], [7], [8]. However, in more recent studies, substantial data have accumulated suggesting that the activation relationship between effector and initiator caspases is less clear than previous studies have suggested. Caspase-6, defined as an effector caspase, possessed significant caspase-8–cleaving activity. Inhibition of caspase-6 activity resulted in the inhibition of caspase-8 cleavage and apoptosis. Therefore, it was concluded that caspase-6 is capable of functioning upstream of caspase-8 to activate caspase-8 activity [9], [10]. Aside from activating caspase-8, caspase-6 was also capable of activating caspase-3, whereas caspase-3 failed to activate caspase-6, suggesting that caspase-6 plays multiple roles in early apoptotic signaling [11]. These results indicate that existing studies reporting on the mechanism and function of caspases activation are far from sufficient.

The best characterized effector caspases are caspase-3 and caspase-7, two key proteases in apoptotic signaling pathways. Both enzymes have similar protein structures, cleave similar substrates, and possess similar apoptotic functions [5], [12]. Caspase-3– or caspase-7–deficient mice on a B6 background are viable, but caspase-3/7–deficient mice have abnormally developed hearts and die immediately after birth [13], [14]. These observations have helped promote a widespread view that caspase-3 and caspase-7 are functionally redundant during apoptosis. While both caspase-3 and caspase-7 are capable of cleaving certain substrates with similar efficiency, such as PARP, RhoGDI, and ROCK I, they also exhibit differences in cleavage patterns for many additional substrates [15]. In other words, caspase-3, a major apoptosis-associated effector caspase, possesses a broader substrate profile than caspase-7 [15].

While the structure, activation, identity of proteolytic substrates, and function of effector caspase during apoptosis are well-known in mammals, little of this information is known in mollusks, including the sequence of effector caspases, and their function in apoptosis. The Pacific oyster *Crassostrea gigas* is a marine bivalve belonging to the phylum Mollusca and is an important cultivated species with the highest production of any cultured aquatic animal species [16]. As a representative of the lophotrochozoa group, *C. gigas* is one of the best-studied in the phylum Mollusca. In a previous study, noradrenaline was capable of inducing apoptosis in *C.gigas* hemocytes, but the number of apoptotic cells under noradrenaline-treated hemocytes were reduced by exposure to the caspase inhibitor Z-VAD-FMK. These results suggested that some members of the caspase family participated during the apoptosis process [17]. Two caspase family members, *Cgcaspase-1* and *Cgcaspase-2*, were cloned from *C. gigas* cDNA [18]. Cgcaspase-1 (GenBank accession number HQ425703) and Cgcaspase-2 (GenBank accession number HQ425706) were annotated by Pfam as an effector and initiator caspase, respectively. Both of those enzymes consisted of a conserved caspase domain and were induced by *Vibrio anguillarum* infection, indicating their probable roles in apoptotic pathways and bacterial defense. These results suggested that oysters contain various caspases, forming a complex apoptotic system.

In this study, we identified a new caspase-like genes, *Cgcaspase-3* in the Pacific oyster, and characterized its protein structural feature. Together with Cgcaspase-1, we subsequently examined the activity of their corresponding expressed recombinant protein and the cell viability of HEK293T cells transfected with Cgcaspase-3-GFP and Cgcaspase-1-GFP. next we investigated and compared the distinct subcellular localization of both caspases. Finally, we compared the mRNA expression pattern of both genes in different tissues and developmental stages.

## Materials and Methods

### Ethics Statement

The Pacific oysters *C. gigas* used in this study were marine-cultured animals and cultured in the aquarium at Institution of Oceanology, Chinese Academy of Sciences (IOCAS). All of the experiments were conducted according to local and national regulations. No specific permissions were required for oyster sample collection and described experiments. All of the field studies were carried out at IOCAS, and did not involve any endangered or protected species.

### Animal Materials and Cell Culture

All of Pacific oysters used in the present study were collected from Qingdao, Shandong province, China, and acclimatized in seawater tanks at 22°C for 7 days before treatment. HeLa and HEK293T cells (ATCC, Manassas, USA) were grown in RPMI-1640 medium (HyClone, Logan, UT, USA) and DMEM/High Glucose medium (HyClone) respectively, supplemented with 10% fetal bovine serum, penicillin (100 U/mL), and streptomycin (100 U/mL), and were maintained at 37°C in 5% CO_2_.

### Cloning cDNA of *CgCaspase-3*

The primers Caspase-3-F1 and Caspase-3-R1 (Table 1) were designed to amplify the middle fragment of *C. gigas Caspase-3* from the oyster cDNA template. Based on the sequence of the middle fragment, gene specific primers were designed for the 3′ and 5′ ends of rapid amplification of cDNA end (RACE). The 3′ end of *CgCaspase-3* was cloned using primer Casp-3F1, Casp-3F2, Casp-3F3, and Oligo(dT)-adaptor (Table 1) according to the user manual of the 3′ RACE system (Invitrogen, Carlsbad, CA, USA). The 5′ end of *CgCaspase-3* was cloned using the primers for Casp-3R1, Casp-3R2, Casp-3R3, and Oligo(dG)-adaptor (Table 1) based on the cDNA with the dCTP tail added by terminal transferase TdT (Invitrogen) following the manufacturer’s instructions.

**Table 1:** All primers used in amplification of *C. gigas* effector caspases.

Primers	Sequence (5′–3′)	Purpose
Caspase-3-F1	CACAATGAGCTAATTGCCGAC	RACE
Caspase-3-R1	GAATCTTTTGGTGAGAGTGGAC	RACE
Casp-3F1	GCGAATGAACCTCAACCCTCTG	RACE
Casp-3F2	CCTCAACCCTCTGTATCCTCCG	RACE
Casp-3F3	GAGTCACGGAAACGAGGATGG	RACE
Oligo(dT)-adaptor	GGCCACGCGTCGACTAGTACT_16_	RACE
Casp-3R1	GTCTGCCACTCGTCATCATAAGC	RACE
Casp-3R2	GACCACGCCCTGAGGAGAC	RACE
Casp-3R3	CGGAGGATACAGAGGGTTGAGGTT	RACE
Oligo(dG)-adaptor	GGCCACGCGTCGACTAGTACG_10_	RACE
cCaspase-1-F	GGACTCAGATCTCGAGATGGAAGAAGCGATGAGCCCAGAGTG	Clone
cCaspase-1-R	GAAGCTTGAGCTCGAGTTTGCTAGTGAAGTAAACCTCCTTG	Clone
cCaspase-3-F	GGACTCAGATCTCGAGATGGCAACCAAAAAGATTCCCCAAG	Clone
cCaspase-3-R	GAAGCTTGAGCTCGAGTTCTGCTAGTTGTTGGGGAGC	Clone
qCaspase-1-F	CTGAACGAGCGGAATGGCA	qPCR
qCaspase-1-R	CGGCGGCTTTCTGTAGTGTA	qPCR
qCaspase-3-F	CGGGAAATTACGGGGAGTTG	qPCR
qCaspase-3-R	TCTTCGGAGGATACAGAGGG	qPCR

### Plasmid Construction and Transfection

The full-length cDNAs of *CgCaspase-1* and *CgCaspase-3* were subcloned into the mammalian expression vector pEGFP-N1 (Clontech, Mountain View, CA, USA). Two *CgCaspase-1–*specific primers (cCaspase-1-F and cCaspase-1-R; Table 1) and two *CgCaspase-3*–specific primers (cCaspase-3-F and cCaspase-3-R; Table 1) were used for PCR amplification of the full-length sequences of two executioner caspases by KOD DNA polymerase (TOYOBO). The PCR products were subcloned into pEGFP-N1, which was linearized by *Xho*-I digestion (New England Biolabs, Ipswich, MA, USA) using the In-Fusion HD Cloning Kit (Clontech) according to the manufacturer’s instructions.

The plasmid transfection experiments were performed using Lipofectamine 2000 (Invitrogen) according to the manufacturer’s instructions.

### Immunoblotting

HEK293T cells were cultured in 6-well plates, grown to ∼80% confluency, and transfected with target plasmid (2.5 µg per well). The cells were washed once with PBS and harvested 24 h after transfection. Immunoblotting analysis was performed with monoclonal antibody GFP (Roche, Penzberg, Germany) and Western Lightning Plus-ECL (PerkinElmer, Waltham, MA, USA).

### Measurement of DEVDase Activity

HEK293T cells were grown in 10-cm culture vessels to ∼80% confluency and transfected with target plasmid (15 µg per well). After culturing for 24 h, these cells were washed once with PBS, harvested, and resuspended in cell lysis buffer. Protein concentration was determined using the Bradford assay [19], using bovine serum albumin (BSA) as the standard. Measurement of caspase-3 activity was performed using the Caspase-3/CPP32 Colorimetric Assay Kit (BioVision, Milpitas, CA, USA) according to the manufacturer’s instructions and the protocol published by Nasirudeen and colleagues [20]. Total protein (50 µg) in 50 µL cell lysis buffer was mixed with 50 µL 2× reaction buffer (containing 10 mM DTT) and 5 µL DEVD- *p*-nitroaniline (*p*NA), and incubated at 37°C for 2 h. Samples were measured by a Varioskan Flash multimode reader (Thermo Scientific, Waltham, MA, USA) at an excitation wavelength of 405 nm.

### Cell Viability Assay

HEK293T cells were seeded in 12-well plates and transfected with target plasmid (1 µg per well) after achieving a confluency of ∼80%. These cells were rinsed once with PBS and harvested 24 h after transfection. Cell suspensions were thoroughly mixed with 100 µL PBS and equal volumes of a 0.4% trypan blue solution (Invitrogen). The numbers of viable and dead cells were counted on a hemocytometer using an inverted phase contrast microscope.

### Confocal Microscopy

HeLa cells were rinsed once with PBS at 24 h after transfection. Next cells were stained with a 2 µg/mL solution of Hoechst33342 (Invitrogen) in PBS for 10 min at 37°C, rinsed twice with PBS and visualized by confocal microscopy.

### Sample Collection

Seven different tissues of six healthy oysters were sampled for RNA extraction, including mantles, gills, gonads, adductor muscles, labial palp, and hemolymph. Four typical larval samples at different developmental stages were collected, including fertilized eggs, D-shaped larval sample, umbo larval sample, and pediveliger larval sample, as well as larval samples at 6, 12, 24, and 48 h after settlement [21].

### RNA Extraction and Transcriptional Analysis of Two Executioner Caspases

Total RNA was extracted from 100 mg samples using 1 mL of Trizol reagent (Invitrogen). cDNA was reverse-transcribed from 1 µg of total RNA in a 20-µL reaction mixture using PrimeScript RT reagent kit with gDNA Eraser (TaKaRa), following the manufacturer’s instructions.

Quantitative PCR was performed in an ABI 7500 Fast Real-Time PCR System (Foster City, CA, USA). Two *CgCaspase-1*–pecific primers (qCaspase-1-F and qCaspase-1-R; Table 1) and two *CgCaspase-3*–specific primers (qCaspase-3-F and qCaspase-3-R; Table 1) were used to amplify products (133 bp and 139 bp, respectively) from oyster cDNA template. Cycling conditions were 95°C for 30 s, followed by 40 cycles of 95°C for 5 s and 60°C for 30 s. A melt curve analysis was then performed at the end of the cycling stage to confirm that one PCR product alone was amplified. After the PCR program, data were analyzed using 7500 software v2.0.1 (Applied Biosystems). The 2^-ΔΔ*C*T^ method was used to analyze the expression level of target genes.

RS18 primers were used as internal control primers for the different developmental stages, following the protocol of Du et al. [22]. Elongation factor (EF) gene expression was used as an internal control for different tissues samples, according to the protocol of Zhang et al. [18].

### Statistical Analysis

Data for all experiments were obtained from experiment run in triplicate and analyzed by one-way analysis of variance (ANOVA) using SPSS (v. 13.0; Chicago, IL, USA). *P*-values <0.05 were considered to be statistically significant.

## Results

### Cloning the Full-length cDNA of *CgCaspase-3*

**Figure 1:**
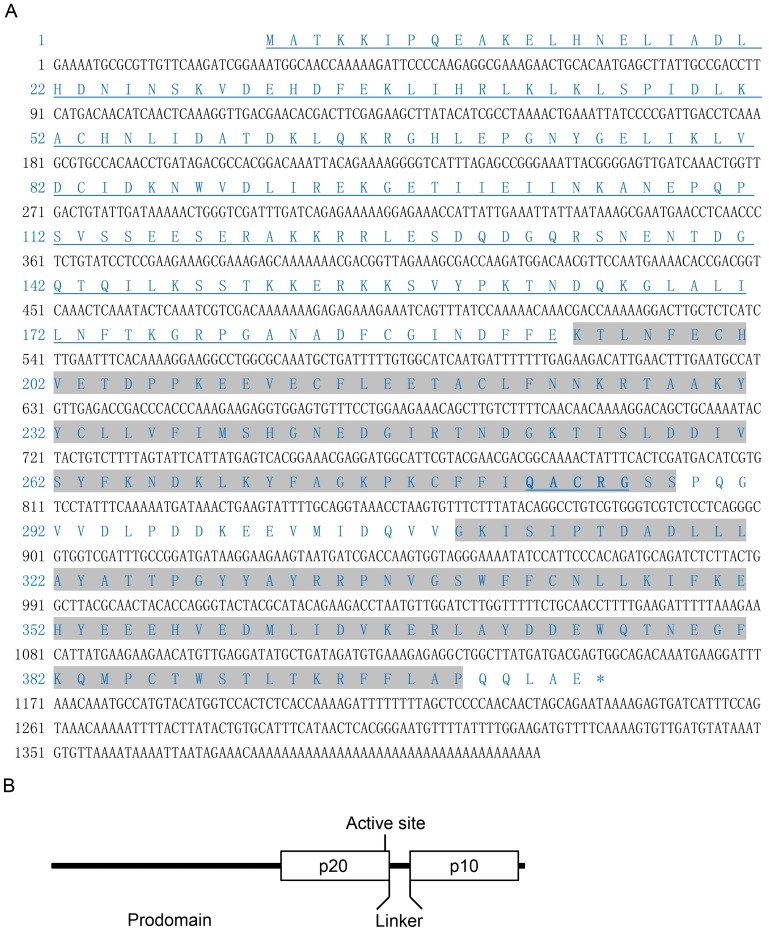
The predicted protein sequence of Cgcaspase-3. (A) The full-length cDNA sequence and deduced amino acid sequence of Cgcaspase-3. Nucleotides and amino acids are numbered on the left-hand side. The prodomain is underlined. The p20 and p10 domains are shaded. The conserved caspase family cysteine active site motif is bold, shaded and underlined. (B) Domains analysis of Cgcaspase-3.

After 3′ RACE and 5′ RACE, we obtained the complete sequence of *Cgcaspase-3*, which consisted of an open reading frame (ORF) of 1215 bp, a 5′ untranslated region (UTR) of 26 bp, and a 3′ UTR of 133 bp with a poly(A) tail. The ORF encodes a predicted protein 404 amino acids in length with a calculated molecular weight of approximately 46.4 kDa and a theoretical isoelectric point of 5.47 (Fig. 1 A and B).

Analysis in the PROSITE database predicted that the Cgcaspase-3 protein contained caspase family p20 (K194-S288) and p10 (G309-P399) domain profiles, and a conserved caspase family cysteine active site motif Q282ACRG (Fig. 1).

### Multiple Sequence Alignment and Phylogenic Analysis

**Figure 2:**
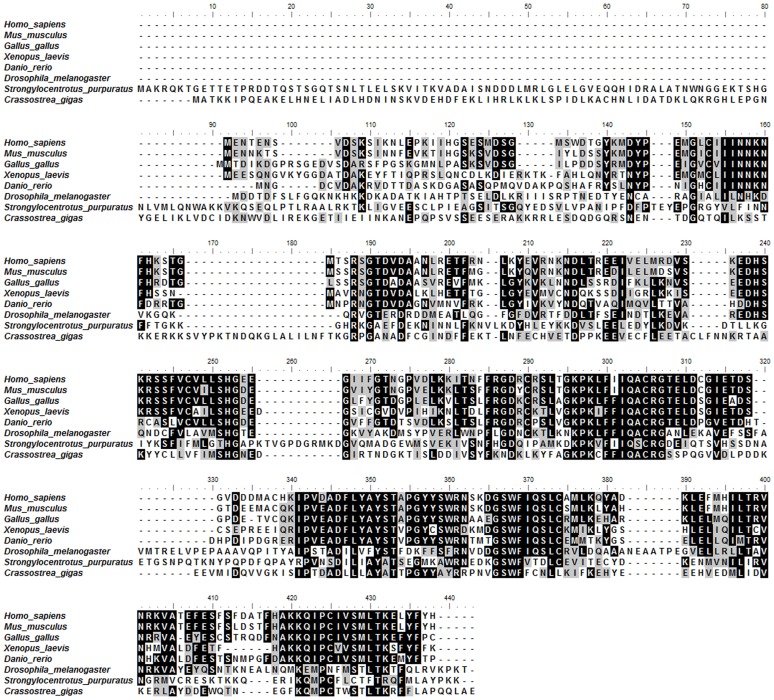
Multiple alignment of the *Crassostrea gigas* caspase-3 (EKC43168) with caspase-3 s from other species. The Cgcaspase-3 protein sequence was aligned with caspase-3 from *Homo sapiens* (CAC88866), *Mus musculus* (AAH38825), *Gallus gallus* (NP_990056), *Xenopus laevis* (NP_001081225), *Danio rerio* (BAB32409), *Strongylocentrotus purpuratus* (XP_003727973), and *Drosophila melanogaster* (AAF55329).

A comparison of the predicted protein sequence of Cgcaspase-3 with other caspase-3 homologs in GenBank revealed that the Cgcaspase-3 sequence displays weak identity with caspase-3 from *Homo sapiens* (25.27%), *Mus musculus* (21.3%), *Gallus gallus* (24.03%), *Xenopus laevis* (21.99%), *Danio rerio* (24.82%), *Strongylocentrotus purpuratus* (21.04%), and *Drosophila melanogaster* (19.16%; Fig. 2). Moreover, sequence alignment overlaps between Cgcaspase-3 and other caspase-3 homologs were primarily localized to the p20 domain and p10 domain, especially the active site motif QACRG in the p20 domain, while the N-terminal prodomain shared an exceedingly low similarity with other caspase-3 homologs. These results suggest that Cgcaspase-3 may be a novel member of the caspase-3 family and possesses similar functionality to other caspase-3 proteins.

**Figure 3:**
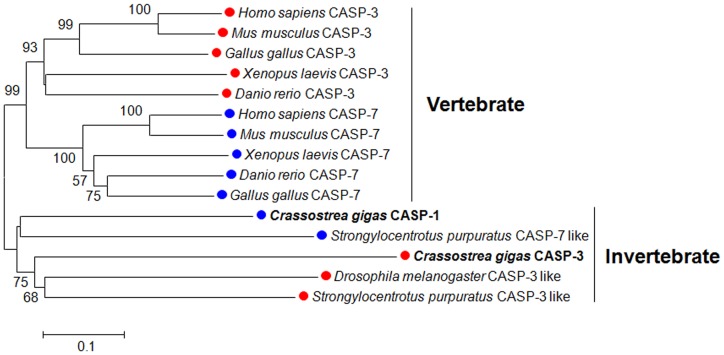
Phylogenic analysis of effector caspases. Consensus neighbor-joining tree with 1000 bootstrap trials by MEGA program, based on the sequences of Cgcaspase-1 (AEB54801) and Cgcaspase-3 from *Crassostrea gigas*, and effector caspases from other species. The caspase-7 s protein of other species were selected from *Homo sapiens* (AAH15799), *Mus musculus* (AAH05428), *Gallus gallus* (XP_421764), *Xenopus laevis* (NP_001081408), *Danio rerio* (AAH95327), and *Strongylocentrotus purpuratus* (XP_789183).

Phylogenetic tree observation showed that vertebrate and invertebrate effector caspases were clustered separately in two distinct groups. Caspase-3 and caspase-7 from vertebrate animals, including *Homo sapiens*, *Mus musculus*, *Gallus gallus*, *Xenopus laevis* and *Danio rerio* were clustered together to form two subclusters. In the invertebrate subgroup, Cgcaspase-3 was first clustered with a caspase-3–like protein from *Drosophila melanogaster* and *Strongylocentrotus purpuratus*, and then grouped together in another subcluster, with Cgcaspase-1 and caspase-7–like protein from *Strongylocentrotus purpuratus* (Fig. 3). These results suggest divergent evolution of the caspase-3 and caspase-7 families between invertebrates and vertebrates.

**Activity assay of recombinant Cgcaspase-1 and Cgcaspase-3 in HEK293 cells.**

**Figure 4:**
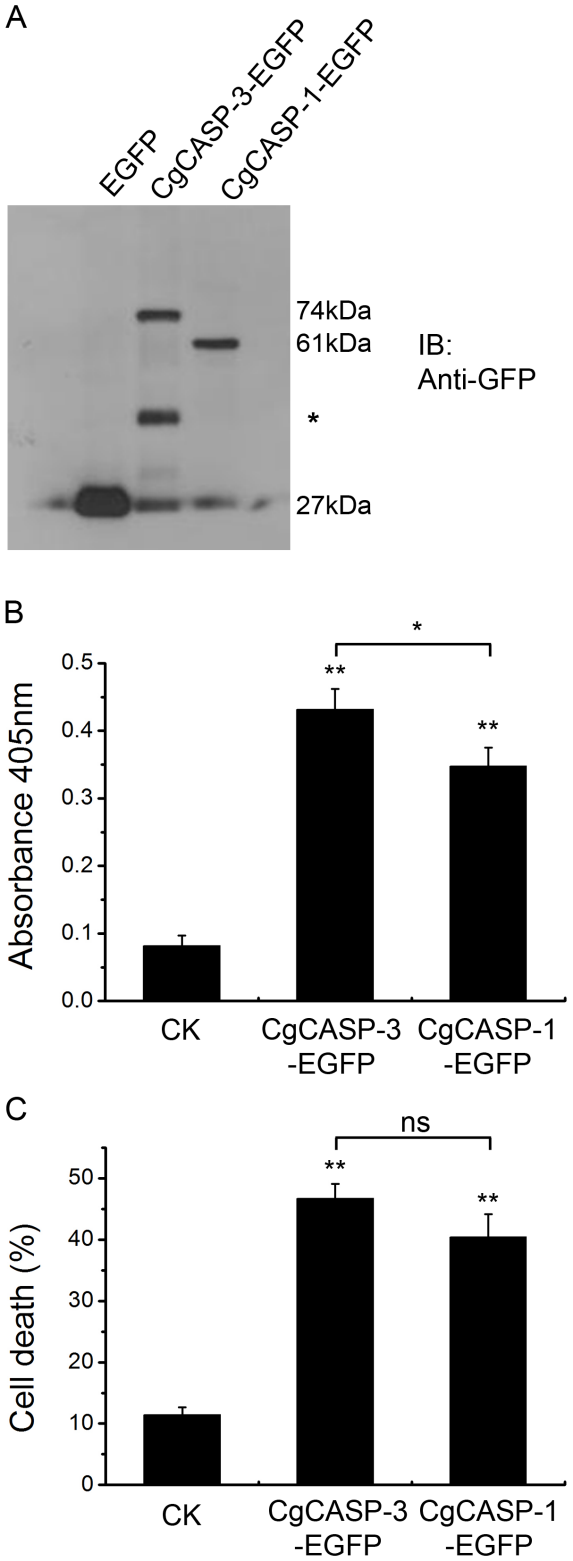
Activity assay of Cgcaspase-3 and Cgcaspase-1. (A) Recombinant expression of both enzymes. The deduced Cgcaspase-3-EGFP protein molecular weight is 74 kDa and the deduced Cgcaspase-1-EGFP protein molecular weight is 61 kDa. The asterisk indicated a non-specific band. (B) DEVDase activity assay of both enzymes. (C) Detection of cell viability with the trypan blue exclusion method. Values are displayed as the mean ± SE of triplicate independent experiments. Differences determined as statistically significant are indicated by asterisks (* *P*<0.05 and ** *P*<0.01); ns, not significant.

To investigate whether both Cgcaspase-1 and Cgcaspase-3 possessed executioner caspase activities, we expressed recombinant Cgcaspase-1 and Cgcaspase-3 as EGFP-tagged fusion proteins in HEK293T cells. The expression levels of both enzymes were detected by immunoblotting using anti-GFP monoclonal antibody. Anti-GFP antibody recognized Cgcaspase-3-EGFP and Cgcaspase-1-EGFP proteins and were identify at their theoretical molecular weight size, suggesting that all proteins were expressed in mammalian cells (Fig. 4A). The activities of Cgcaspase-3 and Cgcaspase-1 were examined through DEVDase activity assay. The DEVDase activities of Cgcaspase-3 and Cgcaspase-1 were 0.432±0.035 and 0.348±0.028, respectively; both significantly higher (*P*<0.05) than the DEVDase activity of EGFP control protein (0.082±0.015; Fig. 4B). These results showed that both Cgcaspase-1 and Cgcaspase-3 catalyzed the cleavage of the *p*NA-labeled tetrapeptide substrate DEVD-*p*NA.

Cell viability was investigated to determined whether Cgcaspase-3 and Cgcaspase-1 in HEK293T cells induced cell death by the trypan blue exclusion method. Percent cell death increased significantly (*P*<0.05) in cells transfected with either pEGFP-N1-Cgcaspase-3 or pEGFP-N1-Cgcaspase-1 compared with that in cells transfected with the pEGFP-N1 empty plasmid control (Fig. 4C). These results suggest that both Cgcaspase-3 and Cgcaspase-1 decreased cell viability and both may be effector caspases in *C. gigas*.

### Distinct Subcellular Localization of Cgcaspase-3 and Cgcaspase-1 in HeLa Cells

**Figure 5:**
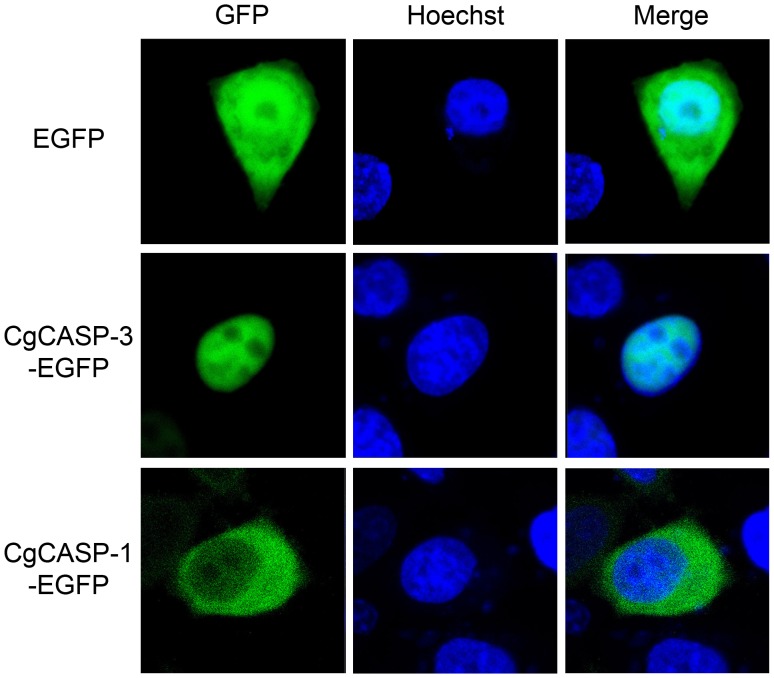
Subcellular localization of Cgcaspase-3-GFP and Cgcaspase-1-GFP in HeLa cells. The left-hand panels depict GFP staining, the middle panels depict Hoechst staining, and the right-hand panels depict merged GFP/Hoechst staining. The upper panels depict localization of the EGFP negative control, the middle panel depicts localization of the Cgcaspase-3-EGFP protein, and the lower panel depicts localization of the Cgcaspase-1-EGFP protein. The green fluorescent signal of Cgcaspase-3-GFP was most strongly focused in condensed nuclei, while the signal of Cgcaspase-1-GFP fusion protein existed primarily in the cytoplasm.

Both caspase-3 and caspase-7 are translated in the cytoplasm; however, caspase-3 plays essential roles in nuclear changes in apoptotic cells, while caspase-7 does not translocate to the nucleus [23]. To directly assess the subcellular localization of Cgcaspase-3 and Cgcaspase-1, both fusion caspases were transfected into HeLa cells and their fluorescent signal were observed with confocal laser scanning microscopy (Fig. 5). We observed that the green fluorescent signal of Cgcaspase-3-GFP was most strongly focused on the condensed nucleus, the signal of Cgcaspase-1-GFP existed primarily in the cytoplasm. The distinct localization of Cgcaspase-3 and Cgcaspase-1 highlighted the functional differences between the two proteins, suggesting that Cgcaspase-3 was a caspase-3–like protein, and Cgcaspase-1 was a caspase-7–like protein.

### Expression Pattern of *Cgcaspase-3* and *Cgcaspase-1* Transcripts At Different Developmental Stages and Different Tissues

**Figure 6:**
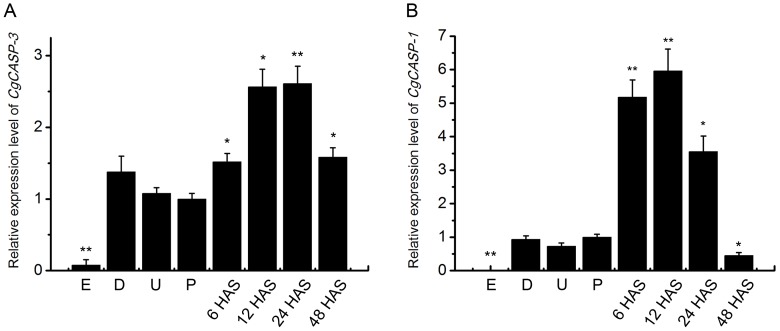
Developmental stage distributions of both effector caspase transcripts. Expression pattern of *Cgcaspase-3* (A) and *Cgcaspase-1* (B) transcripts at different developmental stages. *RS18Q* primers was used as internal control primers and a pediveliger larval sample was used as the reference stage. Dates are displayed as the mean ± SE of triplicate independent experiments. E, eggs sample; D, D-shaped larval sample; U, umbo larval sample; P, pediveliger larval sample; HAS, hours after settlement. Differences determined as statistically significant are indicated by asterisks (* *P*<0.05 and ** *P*<0.01).

The expression levels of *Cgcaspase-3* and *Cgcaspase-1* transcripts were examined using quantitative PCR analysis of total RNA extracted at different developmental larval stages, with *R18Q* used as internal control. We collected larval samples in several typical developmental stages, such as fertilized eggs, D-shaped larvae, umbo larvae and pediveliger larvae. In addition, we collected larval samples at 6, 12, 24, and 48 h after settlement (HAS), because some organs were degraded during this period (Fig. 6A and B). The mRNA of *Cgcaspase-3* and *Cgcaspase-1* were barely expressed in eggs, but stably expressed at D-shaped, umbo and pediveliger larval stages. However, both transcript levels were significantly increased (*P*<0.05) after the larvae settled into the substratum and achieved its peak after 12 h of attachment.

**Figure 7:**
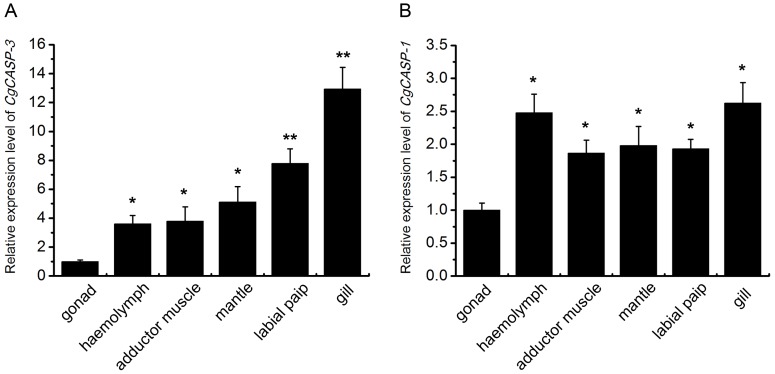
Tissue distributions of both effector caspase transcripts. Expression pattern of *Cgcaspase-3* (A) and *Cgcaspase-1* (B) transcripts in different tissues. *EF* gene expression was used as an internal control and a gonad sample was used as the reference sample. Dates are displayed as the mean ± SE of five independent experiments. Differences determined as statistically significant are indicated by asterisks (* *P*<0.05 and ** *P*<0.01).

The tissue distribution of *Cgcaspase-3* and *Cgcaspase-1* transcripts were examined in healthy oysters, using *EF* as an internal control. The *Cgcaspase-3* expression level in gills was significantly higher (*P*<0.05) than in other tissues; e.g., 12.9-fold higher than in the gonads, which exhibited the lowest level of *Cgcaspase* expression. *Cgcaspase-3* expression in the labial palp, mantles, adductor muscles and hemolymph was 7.8, 5.1, 3.8, and 3.6-fold higher than that of gonads, respectively (Fig. 7A). The *Cgcaspase-1* expression level was highest in gills, but was only 2.6-fold higher than that in the gonads. There was no marked difference in *Cgcaspase-1* transcript levels among the labial palp, mantles, adductor muscles, and hemolymph, which were about 2-fold that of gonads (Fig. 7B).

## Discussion

Caspases are synthesized as catalytically-dormant tripartite proenzymes that consist of a prodomain, a large subunit and a small subunit. Both the large and the small subunits contribute residues to form a substrate-binding domain and make up the active form of the protease, whereas the prodomain varies considerably among different caspases. The prodomains of effector caspases are typically short peptides of unknown function, while the prodomains of initiator caspases are long peptides involved in interactions with other proteins. Caspases have a conserved pentapeptide active site, QACXG (where X is R, Q, or D). The invariant Cys in pentapeptide is a vital residue playing important roles in substrate degradation. Caspases recognize a short tetrapeptide sequence in targeted substrate polypeptides. The last amino acid residue of the tetrapeptide sequence must be an aspartate, after which caspases cleave the peptide sequence, accountings for its appellation as a cysteinyl aspartate-specific proteinase [5]. In previous studies, we obtained an effector caspase sequence in the Pacific oyster *C.gigas*, which we referred to as Cgcaspase-1 and which lacked the long prodomain [18]. In the present study, we isolated another effector caspase, Cgcaspase-3. The open reading frame of *Cgcaspase-3* contained 1215 nucleotides, predicted to encode a protein of 404 amino acids (Fig. 1). The protein sequence comprised canonical domains, such as prodomain, p20 domain, and p10 domain, found in other known caspase-3 homologs. Sequence analysis indicated that, although the predicted full-length Cgcaspase-3 protein shared only 19.16–25.27% identity with other caspase-3 homologs, the caspase families p20 and p10 domain profiles and conserved pentapeptide active-site motif QACRG shared higher identify with known caspase-3 homologs (Fig.2).

Using the neighbor-joining tree method of phylogenetics, we observed that the branches of effector caspase sequences in vertebrates clustered together. For example, the sequences of caspase-3 and caspase-7 formed subclusters. The invertebrate effector caspases formed another cluster, which was distant from the vertebrate cluster. Invertebrate caspase-3 and capase-7 sequences also diverge into two subgroups. Taken together, the data suggest that vertebrate and invertebrate effector caspases may have evolved from a common ancient gene, and then diverged separately in the invertebrate and vertebrate phyla. A caspase-7 homolog was not found in *Drosophila*; it may have been lost during evolution (Fig. 3).

Considering the low sequence identity among these caspase homologs, it was necessary to validate the executioner caspase activity of Cgcaspase-3 and Cgcaspase-1. A tetrapeptide substrate of effector caspase was labeled with the chromophore *p*NA. When the effector caspase recognized the DEVD, *p*NA was cleaved from DEVD-*p*NA, and its light absorbance could be quantified using a spectrophotometer. Based on this method, both enzymes were expressed in recombinant form in HEK293T cells and subjected to an effector caspase activity assay. HEK293T cells transfected by either Cgcaspase-3-EGFP or Cgcaspase-1-EGFP displayed higher DEVDase activity than the cells transfected by EGFP control. These data suggest that the function of both Cgcaspase-3 and Cgcaspase-1 protease were similar to that of effector caspase in the mammalian apoptotic system (Fig. 4B). To further identify the executioner caspase activity of Cgcaspase-3 and Cgcaspase-1, cells transfected with Cgcaspase-3 or Cgcaspase-1were stained with trypan blue, and both living and dead cells were counted. Trypan blue is a diazo dye that does not pass through the intact cell membrane of living cells, but traverses the cell membrane of dead cells. As shown in Figure 4C, the rates of cell death in transfected HEK293T cells were significantly higher than those in the control group. These results suggest that both Cgcaspase-3 and Cgcaspase-1 possessed effector caspase activity.

Although we detected activity in both effector caspases, the question arises as to why overexpressed pro-Cgcaspases are activated and induced cell death. At least three pathways for caspase activation are known to exist in mammalian cells: recruitment activation, trans-activation, and autoactivation [5]. For effector caspase, trans-activation is the primary pathway to stimulate pro-caspase-3/7 maturation and activation. Under this scenario, upstream initiator caspases cleave and activate downstream effector caspases [6]. However, it was observed that RGD peptides can directly induce the autocatalytic activation of procaspase-3, suggesting that autoactivation was another important pathway to activate effector caspases [24]. Furthermore, purified full-length caspase-3 and caspase-7 in *Escherichia coli* were also activated in instance where the initiator caspase was lacking [15]. These results suggest that recombinant expression of full-length pro-Cgcaspase should be activated in HEK293T cells, where both trans-activation and autoactivation pathways exist. It is also worth noting that, although the Cgcaspase-3 protein sequence displays weak identity with caspase-3 from *Homo sapiens* (only 25.27%), the possibility that Cgcaspases activate HEK293T endogenous caspases cannot be excluded, because they are homologs from different species. However, because Cgcaspases were overexpressed in the HEK293T cells, they are likely responsible for the significantly increased DEVDase activity and cell death.

Caspase-3, which has a broader substrate profile than caspase-7, has proven to be a key mediator of apoptosis in mammalian cells [15], [25], [26], [27]. Although its precursor is localized in the cytoplasm, caspase-3 plays important roles in the nuclear changes observed in apoptotic cells [27], [28]. This indicates that some of the cytoplasmic substrates of caspase-3 translocate into the nucleus after cleavage. For example, caspase-activated DNase (CAD) and apoptotic chromatin condensation inducer in the nucleus (Acinus) have been identified in the cytoplasm, but were observed to translocate into the nucleus prior to induction of apoptosis [29], [30], [31]. However, several additional substrates of caspase-3 that are located in the nucleus have been identified [32]. Therefore, caspase-3, like several of its substrates, also appears to translocate from the cytoplasm into the nucleus after the induction of apoptosis. Kamada et al. [23] reported that active caspase-3 localized to the nucleus in apoptotic cells. Meanwhile, the nuclear translocation of active caspase-3 requires proteolytic activation and substrate recognition. Interestingly, caspase-7 could not be located in the nucleus, although it has many substrates in common with caspase-3 [15]. To investigate whether Cgcaspase-3 and Cgcaspase-1 were redundant with one another or resembled a caspase-3–like protein. we analyzed the subcellular localization of both effector caspases in HeLa cells (rather than oyster primary cells, which are difficult to cultivate), Cgcaspase-3 was localized in the nucleus, while Cgcaspase-1 was localized in the cytoplasm (Fig. 5), as observed in previous studies of caspase-3 and caspase-7 in mammals. Thus, Cgcaspase-3 is a caspase-3–like protein and Cgcaspase-1 is a caspase-7–like protein; the proteins were not redundant effector caspases and potentially play different roles during apoptosis.

Apoptosis plays a critical role in many physiologic processes, such as normal tissue and organ development, as well as homeostasis [6]. Information on the distribution of *Cgcasapse-3* and *Cgcaspase-1* during different developmental stages may offer useful clues in investigating the functions of both effector caspases. *Cgcaspase-3* and *Cgcaspase-1* transcripts could barely be detected in eggs, suggesting that few cells were dead at that time point. The mRNA expression of both genes were stable in the D-shaped larvae, umbo larvae, and pediveliger larvae stages, indicating that both caspase retained constitutive expression to maintain cellular homeostasis. After larval settlement onto the substratum, *Cgcaspase-3* and *Cgcaspase-1 *mRNA expression gradually increased and achieved its peak at 12 h after settlement, suggesting that Cgcaspase-3 and Cgcaspase-1 functions in several tissues or organs that degenerate after the settlement of oyster larvae (Fig. 6). We noted that *Cgcaspase-1* mRNA expression increased at an earlier time point than *Cgcaspase-3*. *Cgcaspase-1* mRNA expression nearly achieved its peak after 6 h of attachment and decreased after 12 h, while *Cgcaspase-3* mRNA expression nearly achieved its peak after 12 h of attachment and decreased after 24 h. These results implied that cytoplasmic substrates cleaved by effector caspases at the settlement and metamorphosis stage occurred at an earlier time point than nuclear substrates in *C.gigas*.

*Cgcaspase-3* and *Cgcaspase-1* mRNA were detected in all of the examined tissues, including mantles, gills, gonads, adductor muscles, labial palp, and hemolymph. The universal expression of *Cgcaspase-3* and *Cgcaspase-1* mRNA indicated that both effector caspases may be essential for most physiological functions in *C. gigas*. The highest expression levels were observed in the gills, indicating that both effector caspases are probably involved in immune or metabolic processes in oyster, because gills are the first tissue type involved in material and energy exchange, where biotic and abiotic stresses are more severe than other tissues. However, the expression levels of *Cgcaspase-3* and *Cgcaspase-1* mRNA were not induced by biotic stresses, such as Ostreid herpesvirus 1 and lipopolysaccharide challenge, and abiotic stresses such as air exposure (data not shown). These results were consistent with our previous transcriptome data [33]. We speculate that the *Cgcaspase-3* and *Cgcaspase-1* mRNA was constitutively expressed in adult oysters, but the induction of pro-caspase activity may be challenging. These hypotheses are worthy of further investigation.
